# HMGB1 Induces Secretion of Matrix Vesicles by Macrophages to Enhance Ectopic Mineralization

**DOI:** 10.1371/journal.pone.0156686

**Published:** 2016-05-31

**Authors:** Qiang Chen, Jun-Jie Bei, Chuan Liu, Shi-Bin Feng, Wei-Bo Zhao, Zhou Zhou, Zheng-Ping Yu, Xiao-Jun Du, Hou-Yuan Hu

**Affiliations:** 1 Department of Cardiology, Southwest Hospital, Third Military Medical University, Chongqing, China; 2 Department of Occupational Health, Faculty of Preventive Medicine, Third Military Medical University, Chongqing, China; 3 Experimental Cardiology, Baker IDI Heart and Diabetes Institute, and Central Clinical School, Monash University, Melbourne, Australia; 4 Department of Out-patient, Naval University of Engineering, Wuhan, China; University of Texas Southwestern Medical Center, UNITED STATES

## Abstract

Numerous clinical conditions have been linked to ectopic mineralization (EM). This process of pathological biomineralization is complex and not fully elucidated, but thought to be started within matrix vesicles (MVs). We hypothesized that high mobility group box 1 (HMGB1), a cytokine associated with biomineralizing process under physiological and pathological conditions, induces EM via promoting MVs secretion from macrophages. In this study, we found that HMGB1 significantly promoted secretion of MVs from macrophages and subsequently led to mineral deposition in elevated Ca/P_i_ medium *in vitro*. Transmission electron microscopy of calcifying MVs showed formation of hydroxyapatite crystals in the vesicle interior. Subcutaneous injection into mice with MVs derived from HMGB1-treated cells showed a greater potential to initiate regional mineralization. Mechanistic experiments revealed that HMGB1 activated neutral sphingomyelinase2 (nSMase2) that involved the receptor for advanced glycation end products (RAGE) and p38 MAPK (upstream of nSMase2). Inhibition of nSMase2 with GW4869 or p38 MAPK with SB-239063 prevented MVs secretion and mineral deposition. Collectively, HMGB1 induces MVs secretion from macrophages at least in part, via the RAGE/p38 MAPK/nSMase2 signaling pathway. Our findings thus reveal a novel mechanism by which HMGB1 induces ectopic mineralization.

## Introduction

Ectopic mineralization (EM) is characterized by inappropriate deposition of calcium phosphate complexes in soft tissues. EM is commonly seen in physiological aging process and several common disorders, including infection, atherosclerosis, diabetes, and chronic renal disease [1]. Based on the mechanism leading to mineralization, EM is conventionally classified into: (a) Metabolic EM which arises from sustained hyperphosphatemia and/or hypercalcemia causing widespread mineral deposition particularly the cardiovascular system, kidneys and articular cartilage; (b) Dystrophic EM which represents a response following tissue/organ insults, such as microbial or viral infection, autoimmune diseases and cancer [2, 3].

Extracellular matrix vesicles (MVs) act to initiate the mineralization process leading to physiological and pathological mineralization [1, 4]. MVs are membrane-bound particles and originate from hypertrophic chondrocytes and osteoblasts during osteogenesis during bone formation [5], or from other cells such as cancer cells, smooth muscle cells or macrophages during EM [6]. MVs initiate mineralization in two phases. Firstly, initial formation of hydroxyapatite (HA) in MVs: the influx of calcium and phosphate via annexins and sodium-dependent inorganic phosphate transporters, respectively, leads to initial mineral accumulation in the form of HA crystals enclose to the inside membrane. Secondly, propagation of mineral in the extracellular matrix: HA crystals grow within MVs until membrane rupture. Once exposed to the extracellular matrix, they act as loci or templates for the formation of new crystals via homologous nucleation [7–9].

High mobility group box 1 (HMGB1) is a nuclear constituent bound to chromatin in almost all eukaryotic cells [10]. HMGB1 acts as a damage associated molecular pattern when released by dying cells or secreted by activated cells [11]. Extracellular HMGB1 has drawn increasing attention with recognition of its role in the pathogenesis of autoimmune and inflammatory diseases [11–14]. Recent studies have also shown that HMGB1 is a critical mediator of thrombosis [15, 16]. In addition, HMGB1 is known as a bone-active cytokine involving in both bone remodeling and EM pathogenesis [17, 18]. There is evidence that HMGB1 accumulates extracellularly in the area associated with macrophages infiltration and calcification in calcific aortic valve stenosis [19]. Moreover, HMGB1 directly mediates the osteoblastic differentiation of human dental pulp cells [20] and calcification process of vascular smooth muscle cells (VSMC) in patients with diabetes [21]. However, it remains unknown whether HMGB1 is involved in MVs secretion that initiates mineral deposition of EM. Thus, we have studied the role of HMGB1 in formation and secretion of MVs leading to EM and explored the signaling mechanism.

## Materials and Methods

### Ethics and animal rights protection

Male C57BL/6 mice were obtained from the Laboratory Animal Center of Third Military Medical University. All procedures were conducted in accordance with animal care guidelines approved by the Animal Ethics Committee of Third Military Medical University. The animal care and procedures were in accordance with the guidelines of the National Institutes of Health. Mice were monitored daily for signs of illness or distress. During the course of this study, no mouse became severely ill or died at any time prior to the experimental endpoint. At the end of experiments, mice were sacrificed by cervical dislocation under anesthesia.

### Culture and stimulation of murine macrophages

Murine macrophage-like cells, RAW 264.7 was purchased from the Cell Bank of the Institute of Biochemistry and Cell Biology (Shanghai, China). Cells were cultured in DMEM medium (Gibco, USA) supplemented with 10% fetal bovine serum (FBS), 200 mM L-glutamine (Gibco) and 1% (v/v) penicillin/streptomycin (Beyotime Biotechnology, Beijing, China) in a 5% CO_2_ humidified atmosphere at 37°C. In experiments designed to induce MVs secretion, cells were treated with HMGB1 (R&D Systems, Minneapolis, MN, USA) in FBS free DMEM containing 0.1% BSA (Sigma-Aldrich, St. Louis, MO, USA) for 24 h. Cells were then incubated in elevated Ca/P_i_ (2 mM CaCl_2_ and 2.7 mM mixture of NaH_2_PO_4_ and Na_2_HPO_4_, pH 7.4) or normal Ca/P_i_ (1.2 mM/0.9 mM) for another 24 h to induce mineral deposition. In mechanistic experiments, cells were pretreated with 5 μM GW4869 (Sigma-Aldrich), 10 μM PD98059, SB203580 or SP600125 (Cell Signaling Technology, Beverly, MA, USA) for 1 h. Dimethylsulfoxide (Sigma-Aldrich) was used as vehicle at a final concentration of 0.1%.

### Isolation of mouse peritoneal macrophages

Mouse peritoneal macrophages were separated and cultured as described previously [6]. Briefly, mice were intraperitoneally (i.p.) injected with 2 ml of 3% thioglycolate medium (Sigma-Aldrich) 3 days prior to harvest. Mice were sacrificed by cervical dislocation, and the peritoneal macrophages harvested by injecting sterile phosphate-buffered saline (PBS) into the peritoneal cavity, gently massaging the abdominal area, and decanting the liquid containing the resident macrophages. After washing with PBS, cells were seeded in a 6-well plate at a density of 1×10^6^ cells/ml in 2 ml serum-free medium. After 2 h, cells were washed with PBS to remove non-adherent cells, and the adherent cells were incubated with medium containing 10% FBS (Gibco) in a 5% CO_2_ humidified atmosphere at 37°C.

### Alizarin Red S and von Kossa staining for calcium

Calcium deposition was assessed by Alizarin Red S staining. After treatment, cells in 24-well plates were fixed with 4% paraformaldehyde at 4°C for 1 h, and stained with 0.2% (w/v) Alizarin Red S (Sigma-Aldrich) for 30 min at room temperature. Excess dye was removed by washing. The extent of calcium mineral deposition is proportional to the amount of Alizarin Red S stain remaining in the culture well. For quantitative analysis, calcium deposition was dissolved in 10% cetylpyridinium chloride (Sigma-Aldrich) under shaking for 1 hour and then measured the absorbance at 405 nm with an Infinite M200 microplate reader (Tecan, Austria). For von Kossa staining, freshly prepared 5% silver nitrate solution was added into the plates which were then exposed to ultra-violet light for 1 h.

### Cell viability assay

Cell viability was assessed using Cell Counting Kit-8 (CCK-8, Dojindo, Kumamoto, Japan), according to the manufacturer’s instructions. Briefly, cells were seeded into a 96-well plate (5×10^4^ cells/well) and then treated with HMGB1 at 0–800 ng/mL for a period of 24 h. After incubation, a CCK-8 solution was added to the medium (1:10 ratio) and incubated at 37°C for 2–4 h. The OD value was measured by the absorbance at 450 nm with an Infinite M200 Microplate reader (Tecan, Austria). Results were expressed relative to the control value as 100%.

### Isolation of macrophage-derived MVs

Cell medium was collected after HMGB1 treatment for 24 h and subjected to centrifugation at 2 000g for 30 min to remove cell debris, followed by 16 500 g centrifugation for 5 min to remove apoptotic bodies and any larger vesicles. Cell-free culture media were transferred to a new tube with addition in 2:1 ratio of the Total Exosome Isolation reagent (Life Technologies, Gaithersburg, MD, USA; Catalog Number: 4478359), well mixed and incubated at 4°C overnight. MV fractions were harvested from the mixture by ultracentrifugation at 10 000 g for 1 h at 4°C (Optima Max Ultracentrifuge, Beckman Coulter, Inc., Indianapolis, IN, USA). MV pellets were resuspended in a convenient volume of 1×PBS and keep at 4°C for up to 1 week, or at ≤-20°C for long-term storage.

### Particle size measurement

MV size was determined by a dynamic light scattering method using Data Transfer Assistance (DTA) software by Zetasizer Nano ZS90 (Malvern Instruments, Malvern, UK) at 25°C.

### Flow cytometry

Flow cytometry was performed by FACSVerse^™^ Flow cytometer (BD Biosciences, San Jose, CA, USA). Firstly, calibration beads (Bangs Laboratories Inc., Fishers, IN) with a diameter of 0.2, 0.5 or 0.8 μm, respectively, were used to adjust settings for detection of MVs. MVs preparation were labeled with FITC-conjugated Annexin V, before acquisition, two fluorescent counting beads (0.5 μm and 3 μm) were added into samples. MVs were defined as Annexin V positive events with the appropriate size distribution (≤ 0.5 μm). Calibration beads of 3 μm at 1×10^5^ particles/ml were used for quantifying the number of MVs. Samples treated with the calcium-chelating agent ethylenediaminetetraacetic acid (EDTA, 20 mM) served as a negative control for annexin V gating. Fluorescence dot plots were analyzed and reconstructed with FlowJo software (Tree star Inc., Ashland, OR). Each experiment was performed at least in triplicates.

### Tissue non-specific alkaline phosphatase (TNAP) activity

TNAP activity was measured colorimetrically by using a TNAP assay kit (Beyotime Biotechnology). MVs were isolated as described above. To reduce residual MVs with cells, cells were washed twice with PBS, scraped and transferred into centrifuge tubes. Cells were pelleted by centrifugation at 300g for 5 min and washed three times with PBS. Cell or MV pellets were homogenized with 1% Triton X-100 in 0.9% saline on ice and centrifuged at 20 000g for 15 min. The supernatant was harvested and incubated with 10 mM Para-nitrophenyl phosphate (pNPP) in 0.1 M glycine-NaOH (pH = 10.3) at 37°C for 30 min. The reaction was stopped by addition of 0.25N NaOH. The absorbance was measured at 410 nm using an Infinite M200 Microplate reader (Tecan, Austria). Values were normalized by protein content assessed by the Bio-Rad protein assay reagent (Beyotime Biotechnology) and TNAP activity was expressed as IU/g protein.

### Transmission electron microscopy (TEM) analysis of MVs

MVs were isolated from the culture supernatants of RAW264.7 cells after treatment with HMGB1 (800 ng/ml) for 24 h in FBS free DMEM containing 0.1% BSA, followed by incubation in elevated Ca/P_i_ medium for another 24 h. Nickel grids with 200 mesh, formvar, carbon coating, and freshly glow discharged were used for the negative staining of MVs. Each grid was placed on a 20 μl sample (approximately 1 mg/ml in PBS) for 10 min, and then washed with ddH_2_O. After washing, grids were negatively stained with phosphotungstic acid and imaged on a JEOL 1400 TEM equipped with a side mount Gatan Orius SC1000 digital camera (JEOL Ltd, Tokyo, Japan).

### MVs-Collagen mineralization assay

MVs-collagen mineralization assay was performed as previously described [22]. Briefly, 96-well culture plates were coated with solution containing 0.01% type I collagen (Sigma) in and 0.1 M acetic acid at room temperature for 4 h, which yielded approximately 8–10 μg/cm^2^ coating. MVs isolated as above were added in equal concentrations (20 μg/well) to type I collagen-coated culture wells in the presence of calcifying medium (DMEM with 10 mM β-glycerophosphate) at 37°C for 3 days. Calcium deposition was assessed by Alizarin Red S staining as described above.

### MVs mineralization in the subcutaneous tissues

Control-MVs or HMGB1-MVs (50 μg) were injected subcutaneously in either side of the mice dorsal area under anesthesia. After the injection, mice recovered from anesthesia and were placed in home cages. Seven days afterwards, mice were sacrificed using chloral hydrate to minimize suffering, and the subcutaneous tissues at the injection sites were dissected, paraffin embedded, and sectioned. The presence of calcium deposits in the subcutaneous tissues was confirmed using von Kossa staining and H&E staining.

### Assay of sphingomyelinase activity

Activities of aSMase and nSMase were measured using an Amplex Red sphingomyelinase assay kit (Molecular Probes, Waltham, MA USA; Catalog Number: A12220) according to the procedures described by the manufacturer. Briefly, after treatment, the cells were washed twice with cold PBS and extracted for 30 min on ice in a lysis buffer containing 1% TritonX-100, 1 mM EDTA. After centrifugation at 20 000g for 30 min at 4°C, the resulting supernatants were diluted in 1×reaction buffer for nSMase analysis (pH 7.4) or in 50 mM sodium acetate for aSMase analysis (pH 5.0). Each reaction contained 50 μg/100 μl cellular extracts, 50 μM Amplex Red reagent, 1U/ml horseradish peroxidase (HRP), 0.1U/ml choline oxidase, 4 U/ml alkaline phosphatase, and 0.25 mM sphingomyelin in 1×reaction buffer. Reaction buffer without sphingomyelinase was used as a negative control. Reaction tubes were incubated at 37°C for 1 h. Fluorescence was measured using Infinite M200 Microplate reader with excitation at 560 nm and emission at 590 nm. The results were expressed as fold change of the control value.

### RNA isolation and RT-qPCR

Total RNA from cells was extracted using the RNAiso Plus (Takara Bio Inc., Otsu, Japan). The cDNAs were synthesized by PrimeScript^™^ RT reagent Kit with gDNA Eraser (Takara). Expression of the genes of interest was examined through a Bio-Rad CFX96^™^ Real-Time System with SYBR^®^ Premix Ex Taq^™^ II (Takara). GAPDH was used as an internal control in quantitative analysis. Primers used in real-time PCR analyses are listed in S1 Table. PCR amplification protocol involved 40 cycles of denaturation at 95°C for 10 seconds, primer annealing at 59°C for 20 seconds and primer extension at 72°C for 10 seconds. Each sample was analyzed at least in triplicate. The threshold cycle number (Ct) values of genes were determined. Gene expression level was normalized to GAPDH and presented as the fold change (2^−ΔΔCt^) above control group.

### Western blot analysis

Cells from different experimental conditions were harvested and lysed in RIPA buffer (Beyotime Biotechnology), which contained a cocktail of both protease and phosphatase inhibitors (Roche, Basel, Switzerland). Protein concentration was determined by the BCA protein assay (Beyotime Biotechnology). Samples containing 30 μg of protein were subjected to 10% SDS-PAGE. After protein transfer to nitrocellulose membranes, the membranes were blocked and incubated with various primary antibodies at 4°C overnight. The primary antibodies used were phospho-Erk1/2 (Thr185/Tyr187), Erk1/2, phospho-p38 MAPK (Thr180/Tyr182), p38 MAPK, phospho-JNK (Thr183/Tyr185), JNK (Cell Signaling Technology Inc.). β-actin or GAPDH (Sigma) was used as a loading control. The special Odyssey secondary antibodies were IRDye680 donkey anti-rabbit antibody or IRDye800 donkey anti-mouse IgG antibody. The fluorescent signals were detected and quantified using an Odyssey Infrared Imaging System (LI-COR, Lincoln, NE, USA).

### Immunofluorescence assay

After treatment with HMGB1 (800 ng/ml) for 24 h, RAW264.7 cells were rinsed briefly with cold PBS and fixed with 4% paraformaldehyde for 30 min at room temperature. After washed with PBST (PBS + 0.1% Tween 20), cells were permeabilized with 0.25% Triton X-100 for 10 min, incubated with 1% BSA in PBST for 1 h to block non-specific binding of the antibodies, and then incubated with primary antibody anti-Runx2 (rabbit, 1:100, Santa Cruz Biotechnology, Inc), or OPN (goat, 1:100, Santa Cruz Biotechnology, Inc) overnight at 4°C. After washing with PBST, cells were incubated with secondary antibody (Alexa Fluor^®^ 488 donkey anti-rabbit IgG, 1:100, Invitrogen) or (Alexa Fluor R488 chicken anti-goat IgG, 1:100, Invitrogen) for 1 h at 37°C. Nuclei were labeled with DAPI. Replacing the primary antibody with a normal IgG served as a negative control. Images were collected using a Zeiss LSM 780 confocal microscopy (Carl Zeiss, Jena, Germany) for detection of Runx2 and OPN expression.

### RNA interference

Small interfering RNA (siRNA) was used to suppress specific mRNA expression in RAW264.7 cells. Briefly, 80 nM TLR2 siRNA, TLR4 siRNA, RAGE siRNA or negative siRNA was introduced into the cells with siRNA transfection reagent in siRNA transfection medium. Transfection efficiency was monitored by the gene expression of TLR2, TLR4 or RAGE. Confluent cells were used 2 days after transfection. All regents used in RNA interference were purchased from Santa Cruz Biotechnology, Inc.

### Statistical analysis

Data analysis was performed using the GraphPad Prism 5.0 software package (San Diego, CA, USA). All experimental data are expressed as the mean ± standard error of the mean (SEM) with each experiment repeated at least three times, unless otherwise stated. The data comparisons were performed using Student’s *t*-test or one-way analysis of variance (ANOVA), and *P*<0.05 was considered statistically significant.

## Results

### HMGB1 induces mineralization of RAW264.7 cells in culture

**Fig 1:**
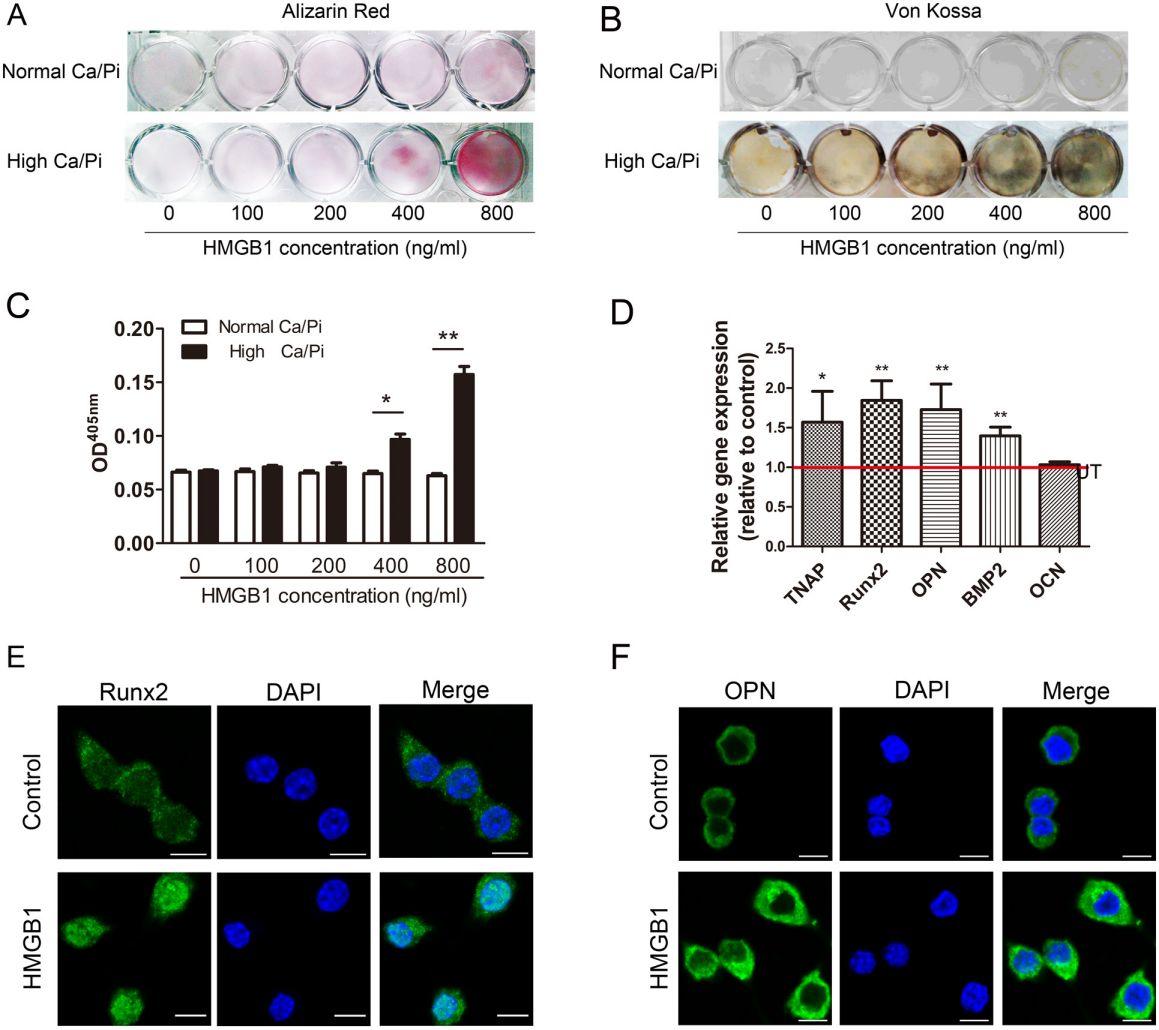
HMGB1 promotes matrix mineralization of RAW264.7 cells. Calcium deposit was visualized with Alizarin Red staining**(A)** and von Kossa staining **(B)**, and Alizarin Red stains were eluted and measured at 405 nm **(C)**. *P <0.05; **P <0.01, versus normal CaPi groups (n = 3). **(D)** Effects of HMGB1 on the mRNA expression of the mineralization-related markers *TNAP*, *Runx2*, *OPN*, *BMP2* and *OCN*. The results were expressed as fold change of the untreated (UT) groups. **(E** and **F)** Effects of HMGB1 on the protein expression of the Runx2 and OPN detected by immunofluorescence staining. (Bar = 10 μm).

To test the effects of HMGB1 on mineralization of RAW264.7 cells, cells were treated with 0800 ng/ml HMGB1. A significant increase in calcium deposition was observed following HMGB1 treatment at 400 and 800 ng/ml under high Ca/P_i_ conditions as determined by Alizarin Red staining and von Kossa staining. However, no calcium deposition was observed under normal Ca/P_i_ conditions (Fig 1A–1C). HMGB1 (800 ng/ml) also significantly increased mRNA expression of the mineralization-related markers *TNAP*, *runt related transcription factor 2 (Runx2)*, *osteopontin (OPN) and bone morphogenetic protein-2 (BMP2)* (Fig 1D). Comparable changes in Runx2 and OPN protein expression were also observed by immunofluorescence staining (Fig 1E and 1F). In addition, HMGB1 (0–800 ng/ml) treatment did not affect the viability of RAW264.7 cells (S1 Fig). These data suggest that HMGB1 directly induces mineralization of RAW264.7 cells in the presence of high Ca/P_i_.

### HMGB1 enhances MVs secretion from macrophages

**Fig 2:**
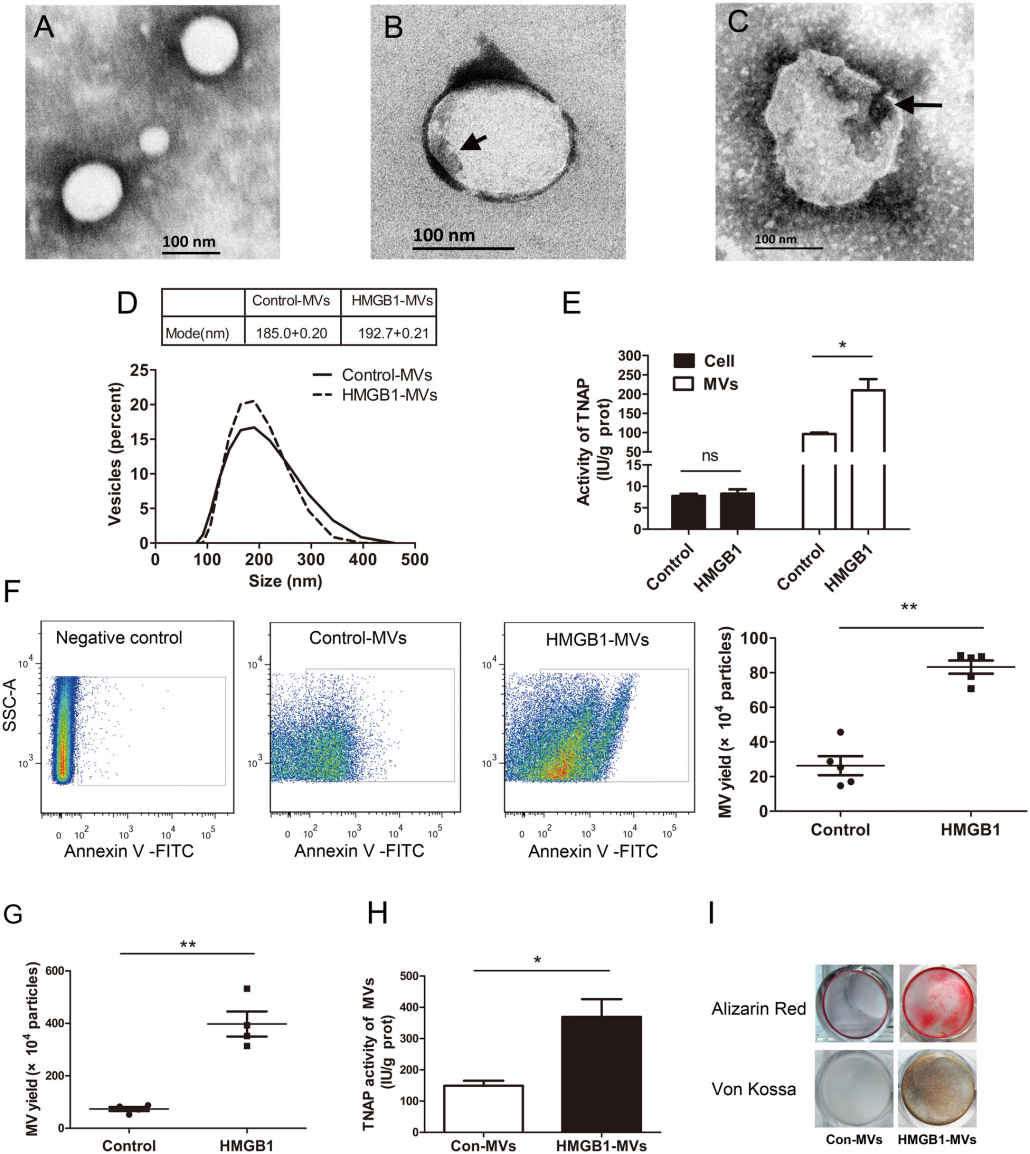
HMGB1 induces matrix vesicles secretion from RAW264.7 macrophages (A-F) or mouse peritoneal macrophages (G-I). Transmission electron microscopic images show MVs with no crystalline hydroxyapatite **(A)**, with early mineralized electron dense materials (short arrow) near the inner leaflet **(B)** and with matured crystal-like structures (long arrow) in the vesicle interior **(C)**. **(D)** Size distribution of MVs was determined by dynamic light scattering method. **(E)** Assay of TNAP activity in cells (filled bar) or MVs (white bar). *P <0.05 (n = 3). **(F)** MVs secretion from cells was determined by flow cytometry. **P <0.01 (n = 5). **(G)** Effect of HMGB1 on secretion of MVs from mouse peritoneal macrophages was determined by flow cytometry. **P <0.01 (n = 4). **(H)** TNAP activity in MVs was determined. *P<0.05 (n = 4). **(I)** HMGB1-MVs from mouse peritoneal macrophages initiated mineralization in type I collagen-coated dishes as determined by Alizarin Red staining and von Kossa staining. For all grouped data, values are presented as the mean±SEM.

MVs released from macrophages have been shown to increase the microcalcification in atherosclerotic plaques [6]. So we investigated whether MVs secretion from macrophages was enhanced by HMGB1. TEM analysis identified MVs derived from HMGB1-treated RAW264.7 cells as membrane-bound vesicles, showing hydroxyapatite nucleation on the inside membrane and within vesicles after incubation in elevated Ca/P_i_ (Fig 2A–2C). Particle size analysis revealed that RAW264.7 cells released a population of vesicles with diameter between 50 to 500 nm and there was no effect of HMGB1 on the size-distribution of MVs (Fig 2D). As shown in Fig 2E, TNAP activity, considered as a marker of MVs maturation [22, 23], was higher in HMGB1-induced MVs. The cellular TNAP activity was <10% of that in MVs and was unaffected by HMGB1 treatment. We quantified secretion of MVs in the supernatants by flow cytometry analysis. Treatment with HMGB1 at 800 ng/ml significantly enhanced MVs secretion (Fig 2F). Furthermore, in mouse peritoneal macrophages HMGB1 also stimulated production of MVs (Fig 2G), TNAP activity in the MVs (Fig 2H), and increased mineral deposits in calcifying condition as revealed by Alizarin Red staining and von Kossa staining (Fig 2I). These results indicate that HMGB1 triggers MVs release by macrophages and TNAP loading into the MVs, which may contribute to mineral deposition in the calcifying conditions.

### HMGB1-MVs initiate mineralization both *in vitro* and *in vivo*

**Fig 3:**
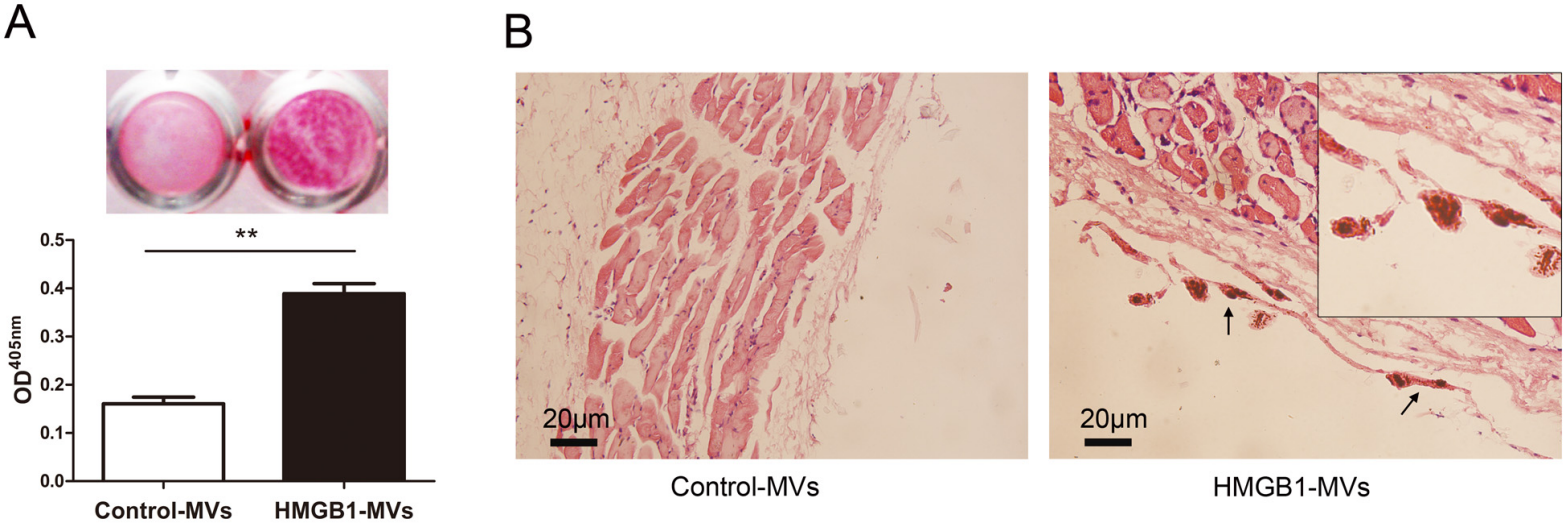
HMGB1-MVs initiate mineralization both *in vitro* and *in vivo*. **(A)** Control-MVs or HMGB1-MVs from RAW264.7 cells initiated mineralization in type I collagen-coated dishes was determined by Alizarin Red staining and stains were eluted and measured at 405 nm. **P <0.01 (n = 3). **(B)** Hematoxylin and eosin (HE) staining of subcutaneous tissue sections 7 days after MVs injection. HMGB1-MVs induced calcium deposits (arrows) were associated with dermal collagen fibers. The presence of calcium deposits was confirmed using von Kossa stain. **P <0.01 (n = 5). For all grouped data, values are presented as the mean±SEM.

Next, we incubated control-MVs (MVs from non-treated cells) or HMGB1-MVs (MVs from HMGB1-treated cells) in type I collagen-coated dishes with calcifying condition. Compared to control-MVs, HMGB1-MVs had increased MVs-collagen mineralization (Fig 3A). To further determine if these MVs could initiate EM *in vivo*, control-MVs and HMGB1-MVs were subcutaneously injected in either side of the mice dorsal area respectively for 7 days. Histological analysis revealed that only HMGB1-MVs induced mineral deposits in the subcutaneous tissues. These deposits were rough in morphology and associated with dermal collagen fibers (Fig 3B). Collectively, MVs from HMGB1-treated cells possess greater potential to initiate mineralization both *in vitro* and *in vivo* compared with that from non-treated cells.

### Neutral sphingomyelinase2 (nSMase2) mediate MVs secretion induced by HMGB1

**Fig 4:**
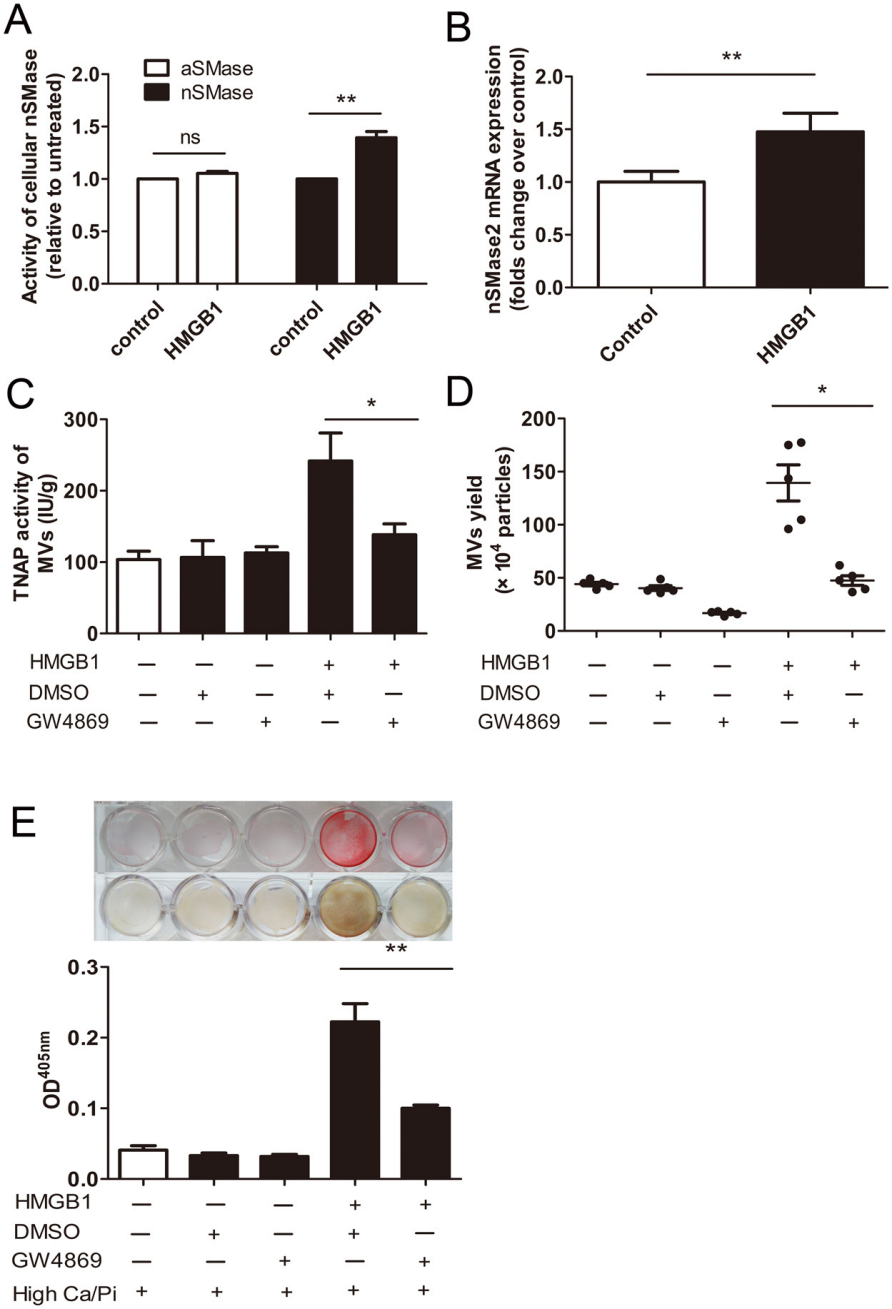
Blocking neutral sphingomyelinase-2 (nSMase) inhibited secretion of matrix vesicles and matrix mineralization induced by HMGB1. **(A)** Cells were treated with 800 ng/ml HMGB1 for 24 h. The activity of nSMase and acid sphingomyelinase (aSMase) of cell homogenate were examined using a sphingomyelinase assay kit. The results are presented as a relative to control group after normalization by total protein content. **P <0.01 (n = 3). **(B)** Levels of nSMase2 mRNA expression were determined by real-time PCR. **P <0.01 (n = 3). **(C**–**E)** Effects of inhibition of nSMase2 with GW4869 (5 μM) on HMGB1-induced MVs secretion and mineralization. TNAP activity in MVs **(C)** and MVs secretion **(D)** was measured. *P <0.05 (n = 5). **(E)** Mineralization was assessed by Alizarin Red staining and von Kossa staining. Alizarin Red stains were eluted and measured at 405 nm. DMSO (0.1%) was used as vehicle control. Values shown are the mean±SEM. **P <0.01 (n = 3).

Since nSMase2 is known as a key signaling molecule regulating vesicles production both in bone mineralization [24, 25] and vascular calcification [26], we examined the effect of HMGB1 on nSMase2 activation. As shown in Fig 4A, activity of nSMase, but not acid sphingomyelinase (aSMase), was upregulated by HMGB1. HMGB1 also enhanced expression of nSMase2 at the mRNA level in RAW264.7 cells (Fig 4B). To examine the role of nSMase2 in MVs secretion and mineralization in response to HMGB1, we inhibited nSMase2 by using the chemical inhibitor, GW4869. Notably, GW4869 dramatically reduced both TNAP activity recovered in MVs pellets and MVs release induced by HMGB1 (Fig 4C and 4D). As expected, these effects were accompanied by an abrogation of mineralization induced by HMGB1 (Fig 4E). These results suggest that HMGB1-induced MVs secretion is mediated by nSMase2.

### p38 MAPK activates nSMase and MV secretion induced by HMGB1

**Fig 5:**
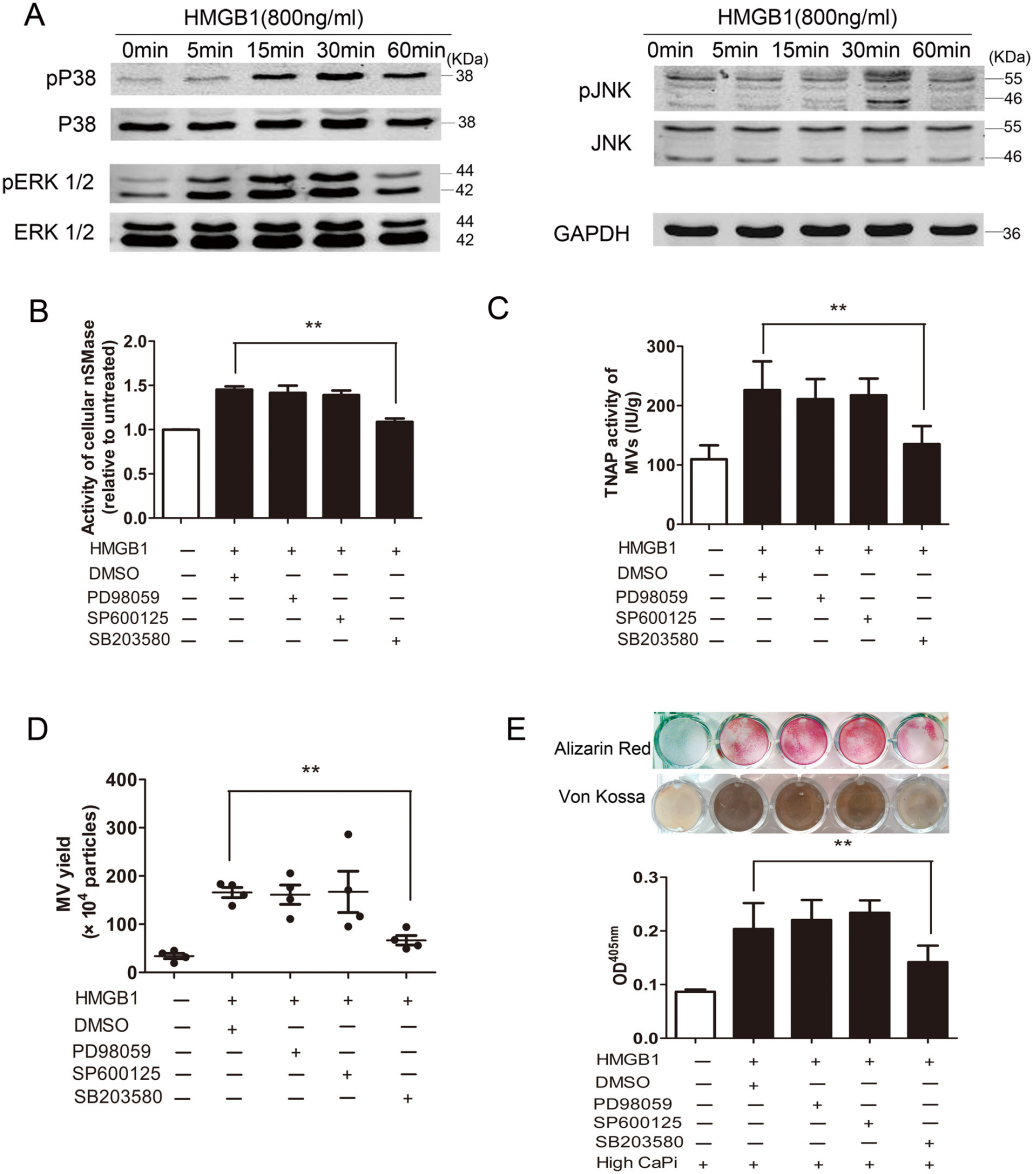
Effects of MAP kinases signaling on HMGB1-induced nSMase2 activation, matrix vesicles releasing and mineralization. **(A)** Cells were treated with 800 ng/ml HMGB1 for 0–60 min. The phosphorylated (upper row) and total (lower row) forms of ERK1/2, p38MAPK and JNK were assessed by western blot analysis. **(B)** Effects of inhibitors for ERK1/2 (PD98059), p38MAPK (SB203580) or JNK (SP600125) on the activity of nSMase. **P <0.01 (n = 3). **(C and D)** MVs were isolated from the culture supernatants, TNAP activity in MVs **(C)** and MVs secretion **(D)** were determined. **P <0.01 (n = 4). **(E)** Mineralization was assessed by Alizarin Red staining and von Kossa staining. Alizarin Red stains were eluted and measured at 405 nm. DMSO (0.1%) was used as vehicle control. Values shown are the mean±SEM **P <0.01 (n = 3).

nSMase2 activation is in parallel to its phosphorylation [27]. The mitogen-activated protein kinase (MAPK) acts upstream of nSMase2 [28], has been suggested to be an important mediator in endothelial microparticles production [29]. So we examined the effects of HMGB1 on the phosphorylation of MAPK proteins in RAW264.7 cells firstly. Immunoblotting revealed HMBG1 treatment for 30 min induced strong phosphorylation of ERK1/2, p38 MAPK and JNK (Fig 5A). To determine which of these MAPKs contributed to nSMase2 activation, effects of specific inhibitors for ERK (PD-98059), p38 (SB-239063) and JNK (SP-600125) were individually tested. Our results showed that only SB-239063, but not PD-98059 and SP-600125, effectively prevented nSMase2 activation induced by HMGB1 (Fig 5B). SB-239063 also suppressed HMGB1-induced TNAP loading into MVs, MVs secretion and subsequent mineralization in elevated Ca/P_i_ conditions (Fig 5C–5E). Taking together, these findings indicate that p38 MAPK mediates HMGB1-induced nSMase2 activation as well as MVs secretion.

### HMGB1 signals through RAGE to promote p38/nSMase-mediated MVs secretion

**Fig 6:**
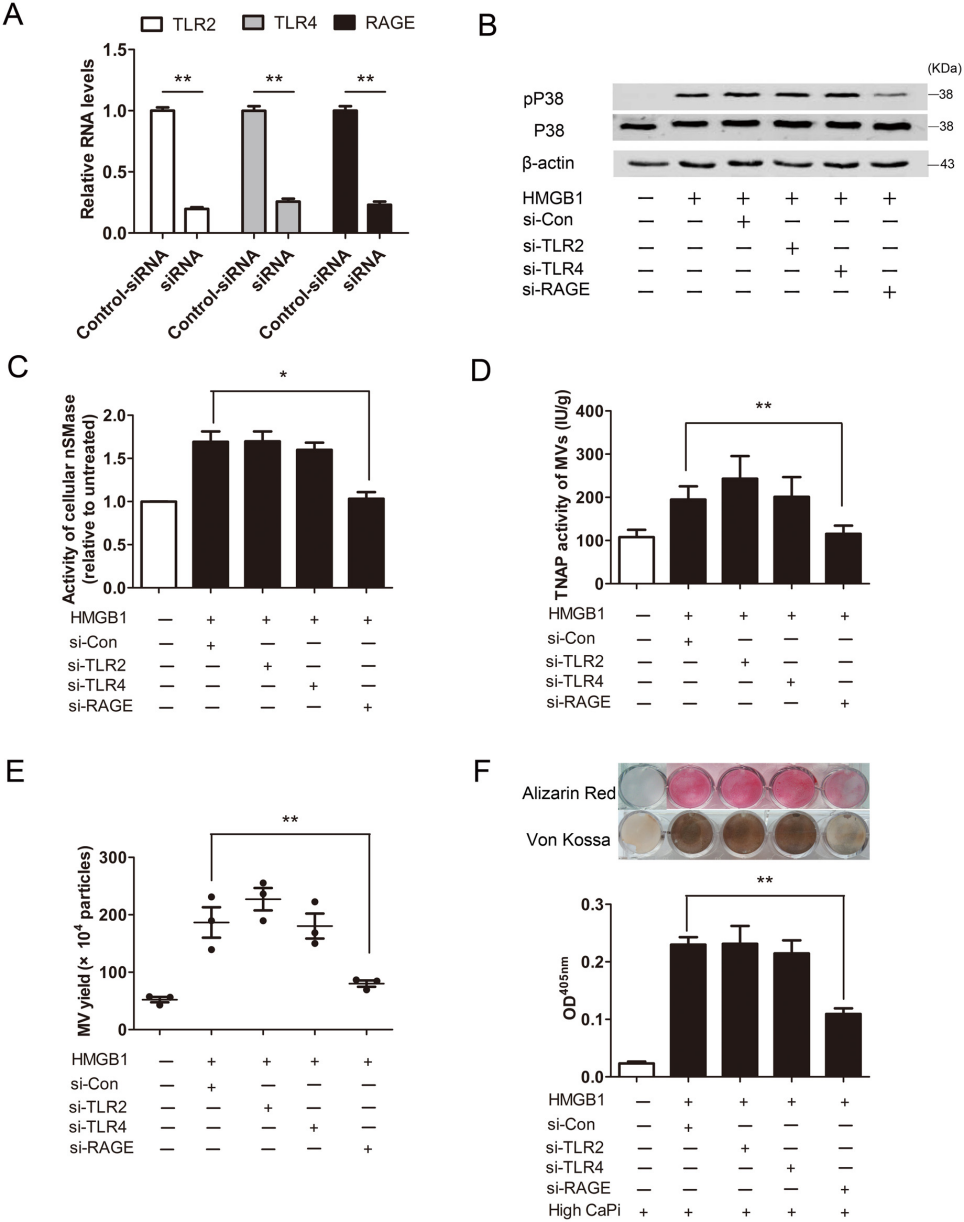
HMGB1 signals through RAGE to promote p38MAPK/nSMase2 mediated secretion of matrix vesicles and mineralization. **(A)** Real-time PCR analysis of mRNA expression in RAW264.7 cells after knockdown of TLR2, TLR4 or RAGE, respectively. **P <0.01 (n = 3). **(B)** Western blot analysis was performed for the p38MAPK phosphorylated forms and corresponding non-phosphorylated proteins. **(C)** nSMase activity of cells were detected at 24 h. *P <0.05 (n = 3). **(D and E)** MVs were isolated from the culture supernatants. TNAP activity in MVs **(D)** and MVs secretion **(E)** were determined. **P <0.01 (n = 3). **(F)** Mineralization was assessed by Alizarin Red staining and von Kossa staining, Alizarin Red stains were eluted and measured at 405 nm. Values shown are the mean±SEM **P <0.01 (n = 3).

Toll-like receptor-2 and -4 (TLR2, TLR4) and RAGE are the principal receptors of HMGB1 on macrophages [30]. To ascertain which type of receptors was involved in the signaling of HMGB1-induced MVs secretion and subsequent mineralization, we knocked down the TLR2, TLR4 or RAGE by siRNA in RAW264.7 cells (Fig 6A). Knockdown of RAGE largely abrogated HMGB1-induced phosphorylation of p38 MAPK and activation of nSMase. TLR2 or TLR4 knockdown, however, did not have such effects (Fig 6B and 6C). In consistent with the effects on p38 MAPK or nSMase, only RAGE-siRNA treatment decreased TNAP activity loading into MVs, prevented MVs secretion and reduced mineralization of RAW264.7 cells in response to HMGB1 (Fig 6D–6F). Thus, RAGE, but not TLR2 or TLR4, is required for HMGB1-induced MVs secretion and subsequent mineralization in calcifying conditions.

## Discussion

**Fig 7:**
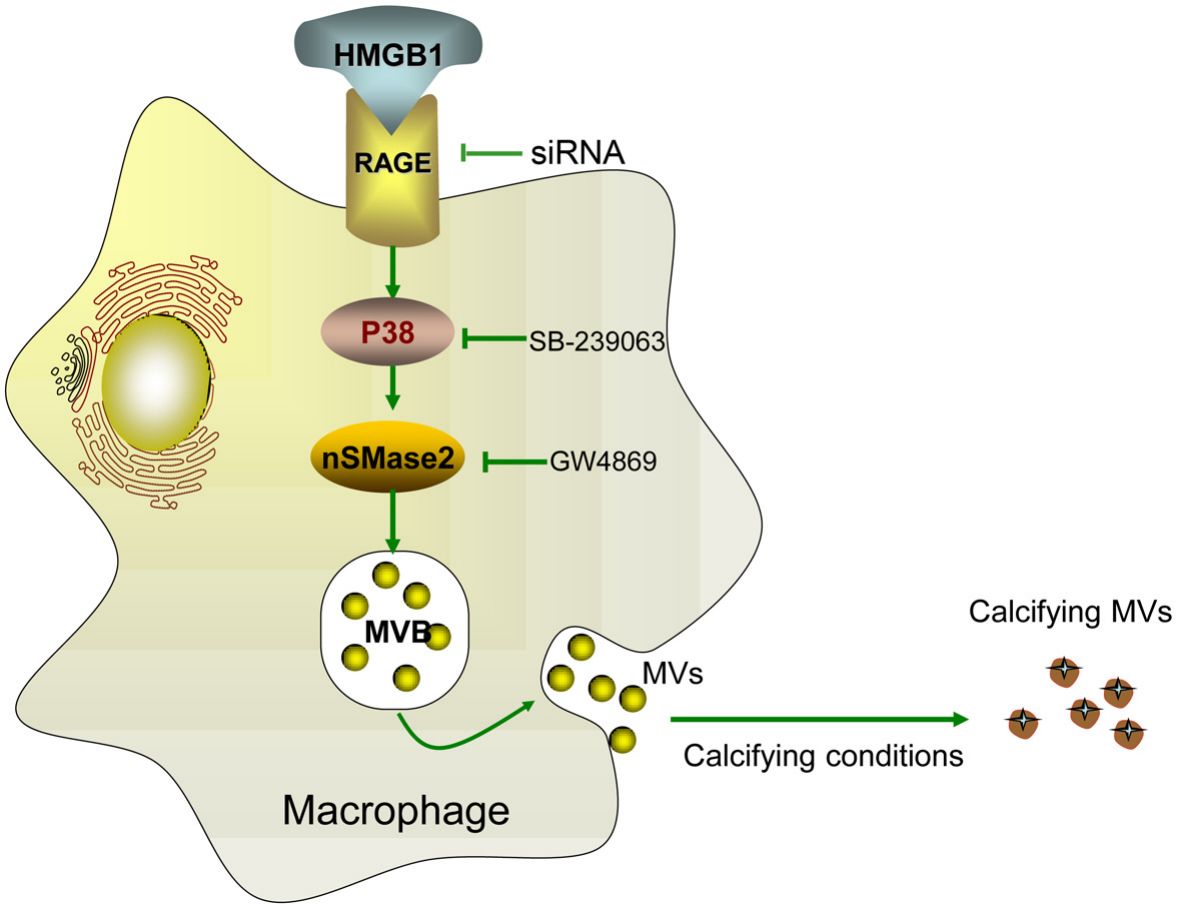
Proposed role of HMGB1 in the secretion of matrix vesicles (MVs) by macrophages and subsequent mineralization. MVs secretion by macrophages can be induced by HMGB1 through activation of neutral sphingomyelinase-2 (nSMase2) by the receptor for advanced glycation end products (RAGE)/p38 mitogen-activated protein kinase (p38MAPK) pathway. Pharmacological or genetic inhibition of RAGE, p38MAPK or nSMase2 is able to impede secretion of MVs. After being released into the extracellular space, MVs shift towards a pro-calcific state in a calcifying-prone condition.

HMGB1, a nuclear protein released from stressed cells, has been indicated as a bone-active cytokine [17] and involved in ossification, arthritis and ectopic mineralization pathogenesis [18, 31]. However, its role in promoting MVs secretion and the mechanism involved remain unknown. The present study provides evidence for the first time that through activating the RAGE /p38/nSMase2 pathway, HMGB1 induces secretion of MVs from macrophages that subsequently participate in mineralization both *in vitro* and *in vivo* (Fig 7).

Elevated extracellular calcium level is known to stimulate the production of calcifying MVs that contain preformed HA crystals and have higher TNAP activity [6, 32]. Besides, some cytokines including platelet derived growth factor (PDGF) and tumor necrosis factor-α (TNF-α) also upregulate MVs secretion while TGF-β, IL-6 and IL-10 have opposite action [6, 26]. Our results reveal that HMGB1 promotes MVs secretion from both RAW264.7 macrophage cell line and mouse peritoneal macrophages, which subsequently leads to mineralization in calcifying conditions. These observations provide a new interpretation on how HMGB1 leads to EM in soft tissues, since HMGB1 is always found to be co-localized with infiltrated macrophage in areas with collagen deposition or mineralization [6, 18]. Flow cytometric characterization further indicates that phosphatidylserine (PS) exposure is enriched on the surface of HMGB1-MVs. This finding is consistent with the reports that MVs derived from calcifying VSMC are rich in acidic phospholipids, such as phosphatidylserine, compared with the corresponding plasma membrane [32]. Phosphatidylserine can bind to annexins (Anx) to form calcium channels in the membrane [33, 34]. More recently, Sophie et al reported that phosphatidylserine can interact with Anx5 and S100A9, forming a PS-Anx5-S100A9 membrane complex, which acts as a nucleation site for the formation of HA [6].

TNAP is one of the best studied MV proteins. Numerous studies have implicated TNAP for its role in mineralization albeit some studies failed to demonstrate the association between high TNAP activity and enhanced mineralization. Such discrepancy might be due to different experimental conditions. Sheen CR et al. recently demonstrated that a selective rise of TNAP expression in the vasculature suffices triggered medial artery calcification [35]. TNAP is enriched on the membrane of MVs and promotes mineralization via hydrolysis of PPi (an inhibitor of biomineralization) to generate Pi [36]. Mice deficient in TNAP function (Akp2^-/-^) display impaired calcification around MVs of growth plate and bone [23]. Thus, TNAP activity is generally regarded as a marker for MVs maturation and pro-mineralization ability [37]. In the current study, we showed that relative to non-treated cells, MVs from HMGB1-treated cells possess a higher TNAP activity and initiate mineralization both in vitro and in vivo. Besides, enzymatic as well as proteomic analyses have identified over 280 proteins in MVs [38]. These include annexins, phospho-1, fetuin-A, and transglutaminase, as well as extracellular matrix metalloprotease (MMP), which are known to be closely related to the function of MVs mediating matrix turnover and mineralization [39–44]. Therefore, it is warranted to further investigate whether and how HMGB1 influences the enzymatic and proteomic signatures of MVs.

Despite the clinical importance of MVs in triggering biomineralization, little has been known on the mechanism regulating MVs biogenesis. nSMase2 has previously been implicated as a key signaling molecule in MVs production and biomineralization [24]. In Fragilitas ossium (fro/fro) mice with deletion of nSMase2 gene, animals display defects in secretion of MVs and impairment in growth plate structures [25]. nSMase2 knockdown in VSMC prevents secretion of calcifying vesicles and subsequent calcification [26]. The present study showed that activation of nSMase2 induced by HMGB1 contributes to secretion of MVs from macrophages, suggesting that nSMase2 also plays a pivotal role in HMGB1-induced mineralization. Intense HMGB1 expression has been observed in areas adjacent to the necrotic core of atherosclerotic lesions contributing to the progression of atherosclerotic plaque [45, 46]. Atherosclerotic plaques containing spotty calcifications have increased share stress, making them more susceptible to rupture [47]. Based on our findings, the presence of abundant macrophages in early microcalcification-rich atherosclerotic plaques imply their importance in intimal calcification. Previous studies have demonstrated macrophages accelerate the osteogenic differentiation of VSMCs via soluble factors including TNF-α [48]. Further investigations are needed to examine the contribution of HMGB1 in atherosclerotic calcification.

HMGB1 is known to signal through the RAGE and TLRs that are expressed by macrophages [10]. RAGE axis has been highlighted as a nodal point in arterial osteochondrogenic mineralization and vascular calcification [49]. Here we demonstrated that HMGB1 signaled through RAGE to promote p38 MAPK/nSMase2-mediated MVs secretion and mineralization. This finding offers a new mechanism for the important role of RAGE axis in biomineralization. A recent study shows that TLR4 mediates mineralization induced by acute phase serum amyloid A in mesenchymal stem cells [50]. Other studies, however, have shown decreased mineralization induced by lipopolysaccharide (LPS) in murine odontoblast-like cells via TLR4/ERK signaling pathway [51]. Interestingly, we found in macrophage cells that TLR2 or TLR4 does not involve in HMGB1-induced mineralization. The different observations may be attributable to differences of stimuli or cell lines used in different experiments.

In summary, we have revealed a novel mechanism by which HMGB1 induces biomineralization. Specifically, we demonstrate for the first time that HMGB1 induces MVs secretion by macrophages and leads to subsequent mineralization *in vitro* and *in vivo*. This is attributable, at least in part, to the activation of signaling pathway involving p38 MAPK and nSMase2 following interaction of HMGB1 with RAGE. Our findings promise further testing of therapeutic interventions to decrease pathological ectopic mineralization by blocking the macrophage derived matrix vesicles secretion induced by HMGB1.

## Supporting Information

S1 Fig Effect of HMGB1 on cell viability of RAW264.7 cells. Cell viability was determined using the CCK8 assay at the range of 0–800 ng/ml tested. Values shown are the mean±SEM (n = 3).
(TIF)

S2 Fig Full image of Fig 1E for RUNX2 staining. (Bar = 10 μm).
(TIF)

S3 Fig Full image of Fig 1F for OPN staining. (Bar = 10 μm).
(TIF)

S1 Table Primers used in real-time PCR analyses. (DOC)
