# Supplementary figures and images for: HMGB1 Induces Secretion of Matrix Vesicles by Macrophages to Enhance Ectopic Mineralization
Source: PLoS One. 2016 May 31;11(5):e0156686. doi: 10.1371/journal.pone.0156686 (PMC4887028; doi:10.1371/journal.pone.0156686)

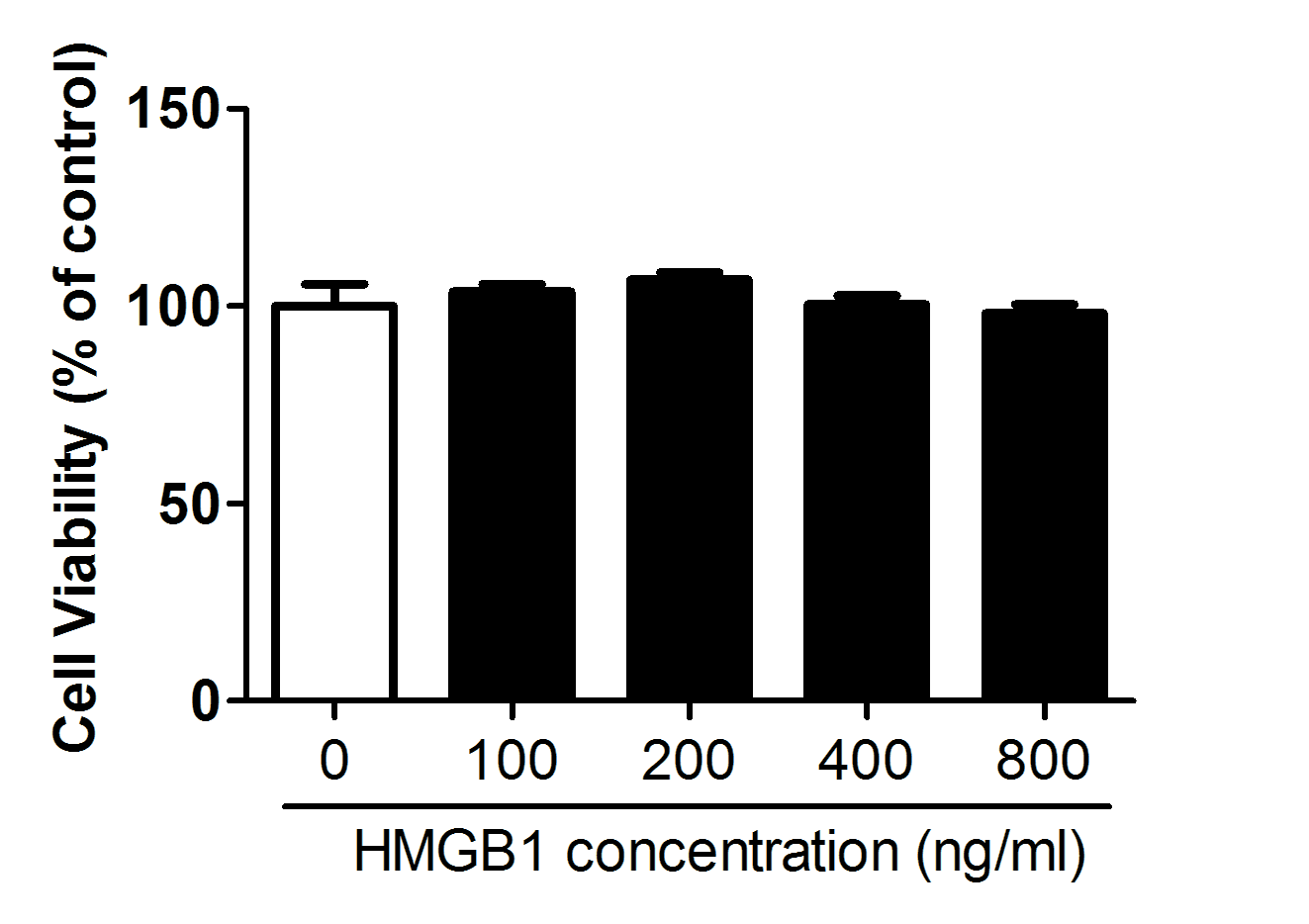

Supplement: S1 Fig — Cell viability was determined using the CCK8 assay at the range of 0–800 ng/ml tested. Values shown are the mean±SEM (n = 3). (TIF) [file pone.0156686.s001.tif]

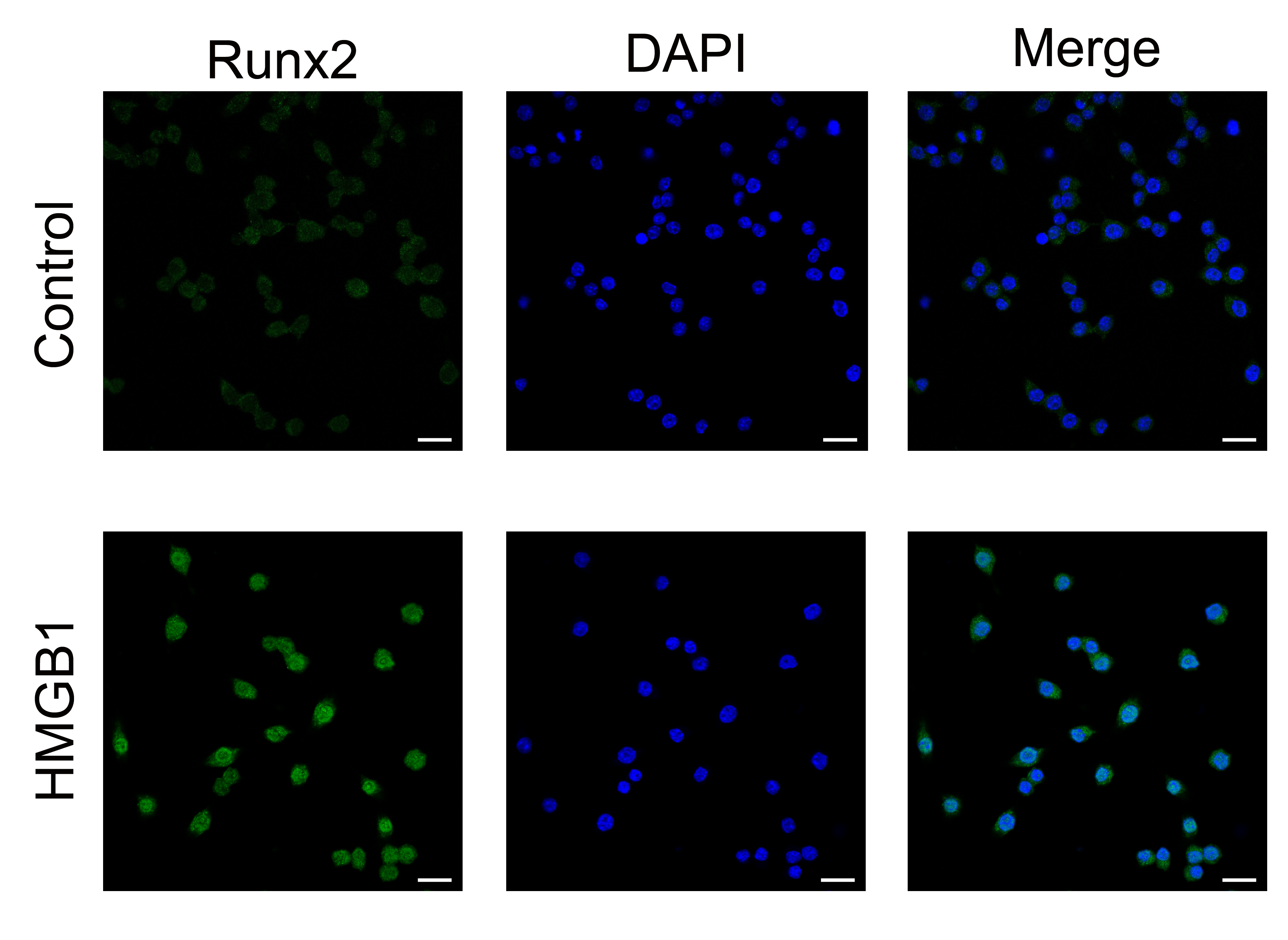

Supplement: S2 Fig — (Bar = 10 μm). (TIF) [file pone.0156686.s002.tif]

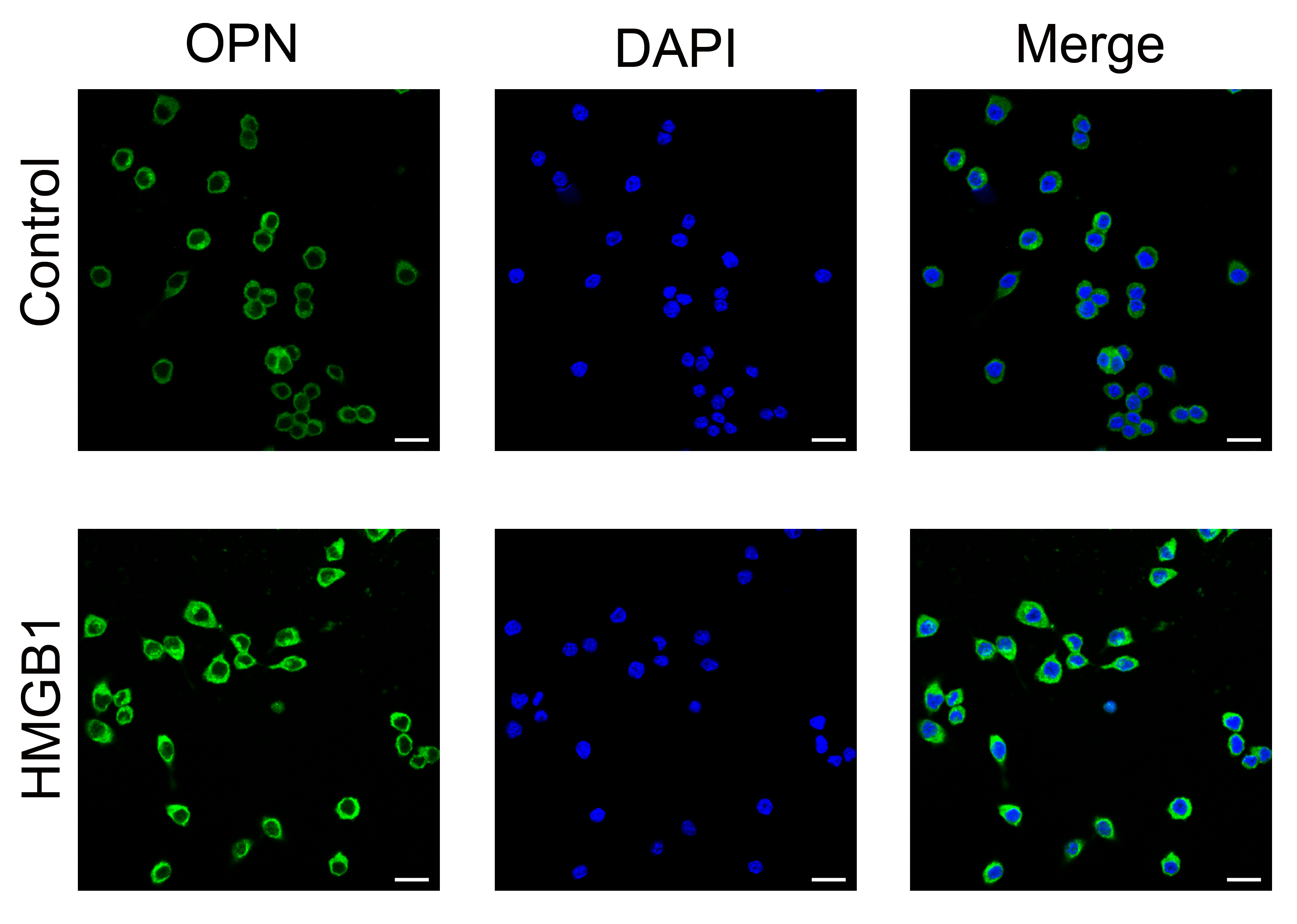

Supplement: S3 Fig — (Bar = 10 μm). (TIF) [file pone.0156686.s003.tif]
