# Supplementary material for: HMGB1 Induces Secretion of Matrix Vesicles by Macrophages to Enhance Ectopic Mineralization
Source: PLoS One. 2016 May 31;11(5):e0156686. doi: 10.1371/journal.pone.0156686 (PMC4887028; doi:10.1371/journal.pone.0156686)
Supplement: S1 Table — (DOC) [file pone.0156686.s004.doc]

### S1 Table. Primers used in real-time PCR analyses.

| Gene | Forward primer | Reverse primer |
| --- | --- | --- |
| nSMase2 | 5′-CTCCAGGTGCTGAGTCCGAG-3′ | 5′-ACCGGG GCTTCCTAGAGAC-3′ |
| Runx2 | 5′-AATTAACGCCAGTCGGAGCA-3′ | 5′-CACTTCTCGGTCTGACGACG-3′ |
| BMP2 | 5′-TGCTTCTTAGACGGACTGCG-3′ | 5′-CTGGGGAAGC AGCAACACTA-3′ |
| Osteocalcin | 5′- GCGCTACCTTGGGTAAGTGG-3′ | 5′-GACCACTCCAGCACAACTCC-3′ |
| TNAP | 5′-AAACCCAGAACACA AGCATTCC-3′ | 5′-TCCACCAGCAAGAAGAA GCC-3′ |
| Osteopontin | 5′-GGCATTCTCGGAGGAAACCA-3′ | 5′-TCCTCTGAGCTGCCAGAAT C-3′ |
| TLR2 | 5′-CGTTGTTCCCTGTGTTGCTG-3′ | 5′-CGTTGTTC CCTGTGTTGCTG-3′ |
| TLR4 | 5′-GCAGTTTCAATCGCATAGAGACAT-3′ | 5′-TGCTTCTGTTCCTTGACCCACT-3′ |
| RAGE | 5′-CGGTGGGTTGAAGGAAGTGA-3′ | 5′-AAGCAATCTCACCCGACCTG-3′ |
| GAPDH | 5′-ATACGGCTACAGCAACAGGG-3′ | 5′-CCTCTCTTGCT CAGTGTCC-3′ |
